# Cryopreservation and post-thaw differentiation of monocytes enabled by macromolecular cryoprotectants which restrict intracellular ice formation†

**DOI:** 10.1039/d5lp00131e

**Published:** 2025-06-09

**Authors:** Natalia Gonzalez-Martinez, Ruben M. F. Tomás, Akalabya Bissoyi, Agnieszka Nagorska, Alexandru Ilie, Matthew I. Gibson

**Affiliations:** a Department of Chemistry, University of Warwick Gibbet Hill Road CV4 7AL Coventry UK; b Manchester Institute of Biotechnology, University of Manchester 131 Princess Street Manchester M1 7DN UK. matt.gibson@manchester.ac.uk; c Department of Chemistry, University of Manchester Oxford Road Manchester M13 9PL UK; d Cryologyx Ltd, Venture Centre, University of Warwick Science Park Coventry CV4 7EZ UK

## Abstract

THP-1 is a monocytic cell line which can differentiate into macrophage and dendritic cells, widely used in immunology. Immune cells are particularly sensitive to cryopreservation, leading to low recovery and/or reduced differentiation capacity compared to non-frozen cells. Current cryopreservation protocols are unsuitable to cryopreserve THP-1 cells in ‘assay-ready’ format, due to the time and resource intensive culturing steps required post-thaw to recover functional cells. We report the cryopreservation of THP-1 cells in vial and multi-well plate format, with significantly enhanced recovery compared to commercial cryoprotectants. This was achieved using macromolecular cryoprotectants (polyampholytes and ice nucleators) which doubled post-thaw recovery relative to DMSO-alone and improved macrophage phenotype post-differentiation comparable to non-frozen controls. Cryo-Raman microscopy demonstrated that the polyampholytes reduced intracellular ice formation compared to DMSO-alone. These results will enable routine banking and ‘assay-ready’ THP-1 cells direct from the freezer, accelerating immunological research.

## Abstract 2

Macromolecular (polymer) cryoprotectants are used to cryopreserve monocytes cells and demonstrate their mode of action is to prevent intracellular ice formation.

## Graphical abstract

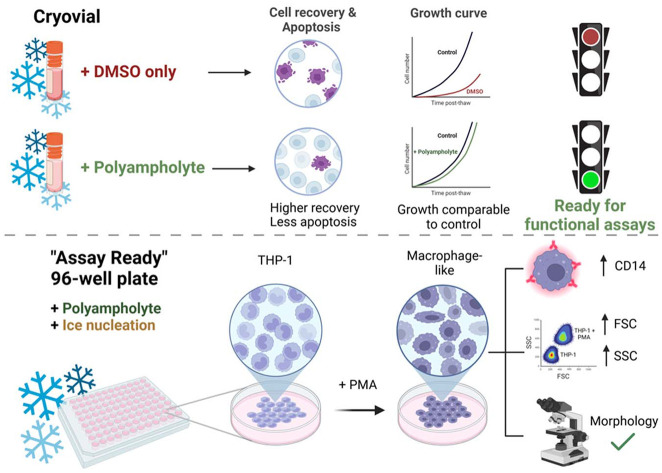

## Introduction

The Tohoku Hospital Pediatrics-1 (THP-1) cell line is derived from human acute monocytic leukaemia. It is widely used to study monocyte, macrophage and dendritic cell biology, immunology and inflammatory disease,^1^ drug-induced cytotoxic responses,^2^ and cell signalling.^3^ These cells are versatile and can be used in co-culture to model diseases.^4^ THP-1s are differentiated into macrophage-like cells using phorbol-12-myristate-13-acetate (PMA), which induces changes in their morphology: from monocyte-like round, single-cells in suspension; to macrophage-like large ameboid-shaped cells that adhere to tissue culture plates. Differentiation is also characterised by the upregulation of macrophage-associated cell markers such as CD14 and CD11b.^5^ Primary human macrophages can be scarce in tissue and challenging to proliferate in culture,^6^ which has prompted the use of macrophages derived from monocytes present in peripheral blood,^7^ but these also require inflammatory mediators for their proliferation and are not present in large numbers (<10%).^8^ In contrast, THP-1 cells are safe to use and fast to grow in standard culture (doubling time: 35 to 50 h).^9^ This makes THP-1 a valuable model for early-stage research.

There have been many developments in the use of THP-1 cells in high-throughput screening within immunology, such as in cytokine secretion,^10^ cell surface detection of immunomodulatory proteins^11,12^ or engineered cell lines.^13,14^ However, a bottleneck in these processes is that these assays require in-house cell expansion. For a typical THP-1 workflow, 1 week to 1.5 month culture time is recommended for optimal cell viability post-thaw.^15^ This is because cryopreservation can severely impact immune cell health and is non-optimised for THP-1 cells.^16–18^ In primary monocytes, low cell recovery is seen post-thaw, and decreases over time, suggesting cryopreservation-induced cell death mediated by apoptosis.^19,20^ However, functionality was not affected,^21^ suggesting that if cryopreservation processes were optimised, workflows could be accelerated, whilst retaining differentiation capacity.^22^ In addition to vial-based cryopreservation (the standard method), cryopreservation in multi-well plates is desirable. The low volumes in 96-well plates (∼100 μL), however, lead to uncontrolled ice nucleation, as water supercools below its freezing point, such that nucleation can occur as low as −20 °C. Ice formation releases heat (as crystallisation is an exothermic process), impacting the cooling rate. This event hinders cellular dehydration after the addition of cryoprotectant, and promotes the formation of intracellular ice, which contributes to low cell viability and recovery. Uncontrolled nucleation also causes ice to form randomly across the 96-well plate,^23,24^ leading to variable outcomes, which would severely impact the accuracy of high-throughput assays. Gibson and Whale demonstrated that macromolecules extracted from pollen (which induce ice nucleation as high as −7 °C (ref. 23 and 25)), improved cell recovery in spheroid cryopreservation, by preserving structural integrity;^26,27^ and in 96-well plates, by decreasing well-to-well variability and maintaining cell function.^25,28^

In addition to the ice nucleation challenge, current dimethyl sulfoxide (DMSO) cryopreservation formulations lead to suboptimal cell recovery. Macromolecular (polymeric) cryoprotectants have emerged as non-permeating extracellular cryoprotectants.^29^ Matsumura introduced polyampholytes – polymers with mixed cationic and anionic side chains – which improve post-thaw cell health in multiple cell types such as mesenchymal stem cells,^30^ and for adherent monolayers^31^ and spheroids.^32^ Previous work suggested (but did not conclusively prove) that these induce cellular dehydration during cryopreservation and mitigate osmotic shock, hence reducing intracellular ice formation and cell death.^33,34^ Macromolecular cryoprotectants which limit ice recrystallisation have also been deployed, benefiting both monolayer and suspension cell cryopreservation outcomes.^35–37^

Here, we report the improved cryopreservation of THP-1 monocytes using 5% DMSO supplemented with a synthetic polyampholyte. Extensive post-thaw characterisation showed improved cell yield and growth, and reduced apoptosis upon addition of the polyampholyte into the cryopreservation medium using cryovials. Cryopreservation in multi-well plates – an ‘assay-ready’ format – was also achieved by the addition of a second macromolecular cryoprotectant: pollen-derived ice nucleators. This minimised well-to-well and biological replicate variability, essential for downstream assays. Cryo-Raman microscopy indicated that the polyampholyte reduced intracellular ice formation, providing a biophysical mechanism of action. Differentiation of the thawed THP-1 cells into macrophages was successfully induced, as microscopy and flow cytometry data showed similar cell phenotype and surface marker expression relative to non-frozen controls. These data will enable the use of cryopreserved THP-1 cells for cell banking and in assay-ready formats, saving time and labour.

## Experimental

### Polyampholyte synthesis

Polyampholyte was synthesised as previously described by Bailey *et al.*^31^ Briefly, poly(methyl vinyl ether-alt-maleic anhydride) with an average *M*_n_ ≈ 80 kDa (10 g) was dissolved in tetrahydrofuran (100 mL) and heated to 50 °C. Once dissolved, dimethylamino ethanol (∼10 g) was added in excess, turning to a pink waxy solid. After 30 min, the waxy solid was dissolved in water (100 mL) and left to stir overnight. The remaining THF was removed under vacuum, and the polyampholyte mixture was purified in dialysis tubing (Spectra/Por, 12–14 kDa MWCO) for 72 h with 6 water changes. The polyampholyte was freeze dried to yield an off-white powder and characterised by NMR and IR, according to previous reports. Chemicals described in this section were purchased from Sigma Aldrich.

### THP-1 cell culture

THP-1 cells (Acute Monocytic Leukaemia derived cell line) were obtained from the AstraZeneca Global Cell Bank and cultured in 75 and 175 cm^2^ cell culture flasks (Corning). THP-1 cells were cultured at 37 °C and 5% CO_2_ in an incubator and maintained at a density between 2 and 9 × 10^5^ cells per mL. Cells were passaged every 3–4 days, with complete media changes happening at least every 7 days. The cell culture media was composed of Roswell Park Memorial Institute (RPMI) 1640 medium (Gibco) supplemented with 10% foetal bovine serum (Sigma Aldrich), 2 mM l-glutamine and 1% antibiotic-antimycotic (Gibco).

### Cell cryopreservation and thawing – cryovials

Cell cryopreservation media consisted of either RPMI 1640 medium containing 2 mM l-glutamine, supplemented with 20% FBS and 5% DMSO; CryoStor® CS5 cell freezing medium (STEMCELL Technologies); or RPMI 1640 medium containing 2 mM l-glutamine supplemented with 5% DMSO, 20% FBS and 40 mg mL^−1^ polyampholyte. Cryoprotectants were sterile filtered using a 0.22 μm syringe filter (Fisher Scientific). After cryoprotectant preparation, THP-1 cells were centrifuged at 100 RCF for 5 minutes and resuspended with the respective cryopreservation solutions, aiming for 1 × 10^6^ viable cells per mL. 1 mL of cell suspension resuspended in cryoprotectant was aliquoted in each cryovial, and these were subsequently introduced in a CoolCell™ LX freezing container (Corning) and into a −80 °C freezer. A pre-freeze count using the trypan blue exclusion assay was performed immediately before cryopreservation to obtain an accurate number for future cell recovery calculations. For short term cryopreservation (<24 h) cryovials were kept in a −80 °C freezer. For longer cryopreservation times, after 24 h, cryovials were stored in liquid nitrogen.

Cryovials were thawed in a water bath at 37 °C for 2 minutes and their contents diluted 1 in 10 with cell thawing media, which contained 20% FBS, 2 mM l-glutamine and 1% antibiotic-antimycotic in RPMI 1640 medium. The cell suspension was subsequently centrifuged at 100 RCF for 5 minutes, the media was decanted, and cells were gently resuspended in the remaining medium (<1 mL). Cell counts were performed using the trypan blue exclusion assay and after obtaining the cell number and viability, the cell pellet was resuspended at the desired cell density, ready for subsequent plating into u-bottom 96-well plates, using fresh pre-warmed cell thawing media. Cell density plated post-thaw depended on the subsequent assays performed and will be specified in their respective subsections.

### Cell cryopreservation and thawing – 96 well plates

To prepare ice nucleator solution, 0.8 g of European Hornbeam Pollen (Pharmallerga) were suspended in 10 mL of sterile water at 4 °C overnight and sterile filtered the following day using a 0.22 μm filter. This solution was mixed at a 1 : 1 ratio with the polyampholyte-containing cryoprotectant solution, consisting of 40% FBS, 10% DMSO and 80 mg mL^−1^ polyampholyte and sterile filtered using a 0.22 μm filter. The final cryoprotectant concentrations were 20% FBS, 5% DMSO, 40 mg mL^−1^ polyampholyte and 50% ice nucleator solution in RPMI 1640 medium. All cryoprotectants were pre-chilled at 4 °C before use.

THP-1 cells were plated at a density of 100 000 cells per well in a u-bottom 96 well plate (Starstedt) and centrifuged at 100 RCF for 5 minutes to remove any media. Subsequently, 100 μL of the final cryoprotectant solution was added per well and a pre-freeze count using the trypan blue exclusion assay was performed on 3 random wells per plate to obtain an accurate number for further cell recovery calculations. The 96-well plate was placed into a CoolSink XT 96-U bottom plate module (Corning) and introduced into a −80 °C freezer for a minimum of 24 h.

To thaw cryopreserved 96-well plates, 150 μL of pre-warmed cell thawing media (20% FBS, 2 mM l-glutamine, 1% antibiotic-antimycotic in RPMI 1640 medium) were added per well. Plates were then placed in an incubator at 37 °C 5% CO_2_ for 12 minutes and centrifuged at 100 RCF for 5 minutes. The cryoprotectant/thawing solution was removed, and plates were resuspended with 100 μL of pre-warmed thawing cell culture media. Cells were incubated at 37 °C and 5% CO_2_ for 24 hours post-thaw, ready for further analysis.

### Cell viability and recovery calculations using trypan blue exclusion assay

At either 0 h (cryovials only) or 24 h post-thaw (cryovials and 96-well plates), an aliquot of cells was diluted 1 : 1 with 0.4% trypan blue (ThermoFisher) and counted under a microscope (Olympus CKX41, 10× objective) using a haemocytometer. To calculate cell viability, the number of cells with intact membranes (unstained cells) were divided by the total number of membrane damaged (cells appear blue) and intact cells counted and expressed as a percentage. To calculate cell recovery, the number of live cells obtained post-thaw (unstained cells) was divided by the cell number frozen (pre-freeze count) and expressed as a percentage.

### Metabolic activity assessment using resazurin reduction assay

After 24 h incubation post-thaw, cells were counted using trypan blue exclusion assay and seeded in U-bottom 96-well plates at several cell densities (1 : 1 serial dilution, 1562 to 50 000 cells per well). Non-frozen control cells were seeded at this timepoint and at the same cell densities for comparison.

To prepare the resazurin solution, ¼ of a resazurin sodium salt tablet (Scientific Lab Supplies) was resuspended in 50 mL of cell culture media without phenol red (all other components were prepared as described above) and sterile filtered using a 0.22 μm filter and syringe. Plates were centrifuged at 100 RCF for 5 minutes at room temperature and cells were resuspended with 100 μL of resazurin solution. The 96-well plate was then incubated at 37 °C, 5% CO_2_ for 4 h. Resazurin to resorufin reduction absorbance was measured using a Synergy HTX Multi-Mode Reader (BioTek) at 570 and 600 nm wavelengths. Resazurin solution alone served as the background empty control well. Each condition was assessed using a minimum of 2 technical and 3 biological triplicates. Experimental results were expressed as percentage resazurin reduction, calculated using the following formula:
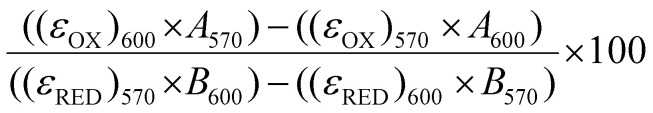
*ε*_OX_ and *ε*_RED_: molar extinction coefficient of resazurin and resorufin at respective wavelengths. *A*_570_ and *A*_600_: sample absorbance at 570 or 600 nm respectively. *B*_570_ and *B*_600_: absorbance of empty control well at 570 or 600 nm respectively.

### Growth curve measurements

After cryopreservation with the different cryoprotectants described in the previous sections, cells were thawed and counted using the trypan blue assay (at 0 h post-thaw for cells cryopreserved in vials, at 24 h post-thaw for cells frozen in plates). 25 000 live cells were seeded per well for every condition in a round bottom 96-well plate (Starstedt). The plates were further divided into different timepoints (24, 48, 72 and 96 hours post-thaw), incubated at 37 °C 5% CO_2_ and counted daily up to 96 h post-thaw. A non-frozen cell control was plated at the same cell density at the 0 h post-thaw timepoint for growth rate comparison. Each condition was assessed using a minimum of 2 technical and 3 biological replicates. To calculate the growth rate and population doubling time of the cells, after every 24 h sampling timepoint, the following formula was used:
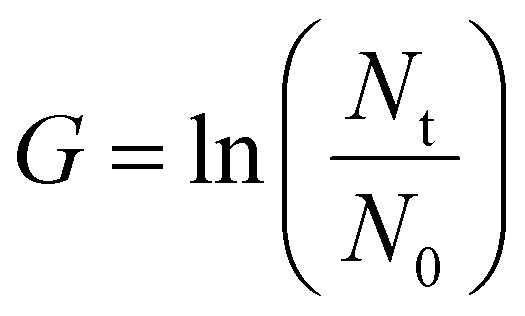

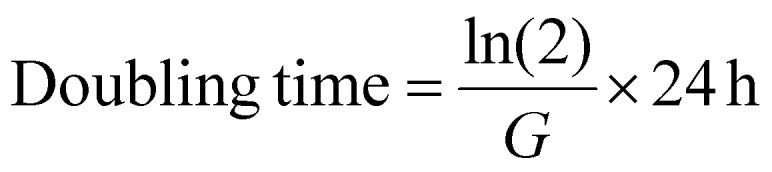
*G* = growth rate, *N*_t_ = cell number after 24 h, *N*_0_ = Initial cell number

### Raman spectroscopy

Raman analysis was performed in a backscattering geometry using an inVia™ confocal Raman microscope (Renishaw) equipped with a cryo-stage (Linkam). A diode laser (532 nm wavelength, ∼20 mW) served as the excitation source, with the laser beam focused onto the sample through a 50× objective lens and a diffraction grating of 600 grooves mm^−1^ (Spectrogon). Spectra were recorded within the range of 50–3800 cm^−1^ and processed using ImageJ software.

### Raman spectroscopy – sample preparation and cooling protocol

THP-1 cells were maintained in cell culture growth media as described in previous sections and upon reaching confluency (1 × 10^6^ cells per mL), cells centrifuged at 300*g* for 5 min and washed twice using DPBS before resuspension with the cryoprotectant solution. The cryoprotectant formulations used were either 5% DMSO, or 5% DMSO + 40 mg mL^−1^ polyampholyte, both in DPBS. Cryoprotectants were formulated in DPBS in the absence of FBS to prevent detection of artifacts. After 10 min incubation in the freezing solution, 1–3 μL of the experimental cell suspension were pipetted or syringed onto the sapphire glass of the cooling stage. The suspension was then gently covered with a quartz crucible (Linkam) to prevent sample evaporation during the experiment. Samples were cooled from room temperature to a final temperature of −50 °C at a cooling rate of 1 °C per minute using a cryo-stage. Once the desired temperature was reached, Raman imaging commenced after allowing 5–10 minutes for the sample to reach thermal and physical equilibrium. Brightfield and Raman spectral images were detected simultaneously. Raman spectra were integrated at each pixel to construct Raman images using characteristic wavenumbers. The spatial distribution of lipids and proteins, representing the size and morphology of frozen cells, was derived from the Raman signal of amide I (1610–1710 cm^−1^). Ice signals were distinguished from other OH stretching signals by analysing the Raman OH stretching band (3087–3162 cm^−1^), its absence denoting no intracellular ice formation, with background subtraction applied to the sides of the peak range as previously reported by Yu *et al.*^52,53^

### Percentage of intracellular ice calculation

Raman images were analysed using a custom Python-based pipeline (code in ESI Methods 1†) to quantify red and blue regions as percentages of the total segmented area. Images were loaded in RGB format (Pillow library) and converted to arrays for pixel-level analysis (NumPy^63^). Threshold-based segmentation was applied, classifying red pixels as high red intensity (≥200) and low green/blue intensities (≤100) and blue pixels as low red (≤100), moderate green (≤150), and high blue (≥100). To account for variability in thresholds, a Monte Carlo simulation with ±10% threshold variation was performed over 50 iterations. Mean percentages and standard deviations were calculated for each region to assess central tendency and variability. Contours of segmented areas were visualized (scikit-image^64^), and annotated images with area percentages were saved using matplotlib,^65^ as shown in ESI Fig. 3–5.† Python version 3.12.1 was used throughout this section.

### Apoptosis analysis

For apoptosis analysis, cells cryopreserved with different cryoprotectants or in different formats (cryovial/96-well plate) were thawed as described in previous sections and divided into 3 different apoptosis timepoints: 4 h, 8 h and 24 h post-thaw. Additionally, unfrozen, untreated cells were plated at the same density and timepoints to act as a negative control of apoptosis. A positive control was created by treating non-frozen control cells with the apoptosis inducer staurosporine (APExBio) at 1 μM concentration for 4 h. At the defined timepoints, plates were prepared for flow cytometry analysis of apoptosis. The FITC-Annexin V/PI apoptosis kit for flow cytometry (Fisher Scientific) was used, and samples were prepared following manufacturer instructions. Cells were incubated with the annexin V/PI for 15 minutes and kept in ice until analysis using flow cytometry.

Unstained samples (negative for both FITC and PI) were classified as “viable”, cells stained only for annexin V-FITC (positive FITC, negative PI) were classified as “early apoptotic”, cells stained for both annexin V-FITC and PI (positive FITC, positive PI) were classified as late apoptotic and cells staining positive for PI only (negative FITC, positive PI) were classified as “late apoptotic/dead”.

### THP-1 differentiation

Plates previously cryopreserved and stored in a −80 °C freezer were thawed as previously described, resuspended in thawing media (containing 20% FBS, 2 mM l-glutamine and 1% antibiotic-antimycotic) and incubated at 37 °C and 5% CO_2_ for 24 hours. Then, each plate was separated into non-differentiated and differentiated THP-1 cells. Each condition was then divided again into low and high density, where low density represents the post-thaw density per well, whereas high density represent several wells pulled together to achieve 100 000 viable cells per well (1 million per mL). Non-frozen controls were plated at this “high” cell density after the 24 h timepoint. The “differentiated” condition wells (including the control) were transferred to flat-bottom 96-well plates to allow adhesion, and treated with 130 nM PMA (Sigma Aldrich) dissolved in growth media containing 20% FBS for 48 h. Then, the PMA-containing media was removed and replaced with regular cell culture media (containing 10% FBS) for 24 h before microscopy and flow cytometry experiments were performed.

### THP-1 differentiation – sample preparation

For flow cytometry measurements of THP-1 differentiation, adherent THP-1 treated with PMA were detached using 5 mM EDTA in DPBS with no Ca^+^ or Mg^+^, after keeping in ice for 15 minutes and gently repeatedly pipetting. Cells were then washed in ice-cold DPBS at 120 RCF for 5 min, then resuspended in ice cold FACS buffer (DPBS + 2% FBS). Fc receptors were blocked by adding 20 μL of Fc Receptor Binding Inhibitor Polyclonal Antibody (ThermoFisher) at 4 °C for 20 minutes. 80 μL of DPBS were then added and, without washing, 1.25 μL of CD14–PerCP Cyanine5.5 (ThermoFisher) were added per 100 μL of sample. This was incubated for 30 minutes at 4 °C, washed twice with FACS buffer, then analysed using flow cytometry.

### Flow cytometry

Flow cytometry was performed using BD Accuri C6 Plus flow cytometer (BD Biosciences). CS&T Research beads (BD Biosciences) were used for instrumental quality control before performing experiments. Green fluorescence (FITC) was detected using the 488 nm excitation laser and 530/30 nm emission filter. Red fluorescence (PI, CD14-PerCP 5.5) was detected using the 488 nm excitation laser and 670 nm LP emission filter. Fluorescence compensation was manually performed to minimise overlap of the FL-1 and FL-3 channels using fluorescence minus one (FMO) control from experimental positive and negative controls. Data analysis was performed using FlowJo (v10.10).

### Phase contrast microscopy

Phase contrast microscopy was performed using an Olympus CKX41 microscope equipped with a XC30 camera using a 10× or 20× objective for a total 100 or 200× magnification. During the THP-1 differentiation experiments, images were taken at 0, 24, 48, 72, and 96 h post-thaw within the 96-well plate. Images were viewed and processed (brightness and contrast only) using ImageJ (version 1.53T). The same settings were used in all microscopy images.

### Statistics and reproducibility

Data analysis was performed using Origin (Version 2024b) and GraphPad Prism (Version 10.3.1). The data was analysed for normality using the Shapiro–Wilk Test. To test for equality of variance between groups, the *F*-test was used. When the data was normally distributed and there were no significant differences in variance between groups, mean comparison between two or more groups were performed using one-way ANOVA and Tukey's *post-hoc* test against the respective control. For comparison between two groups only, two-tailed unpaired *T*-tests were used. Results are reported in these instances as mean ± SD. When the normality or variance tests identified a significant difference, a non-parametric test was used (Kruskal–Wallis with Dunn *post-hoc* test). In this instance, results were visually represented using boxplots, displaying the median and quartiles, and spread was quantified by reporting the values of the quartiles and the interquartile range. All experiments were performed in biological triplicates (independent experiments on different days), and technical duplicates or triplicates (within the same plate) as specified within the respective figure legends to verify reproducibility to ensure consistency across all replicates. Results were considered statistically significant when *P* < 0.05. *P* values in graphs are represented as asterisks meaning: *P ≤ 0.05, **P ≤ 0.01, ***P ≤ 0.001, ****P ≤ 0.0001.

## Results and discussion

### Polyampholyte supplementation in freezing media improves THP-1 monocyte cryopreservation in cryovials

**Fig. 1:**
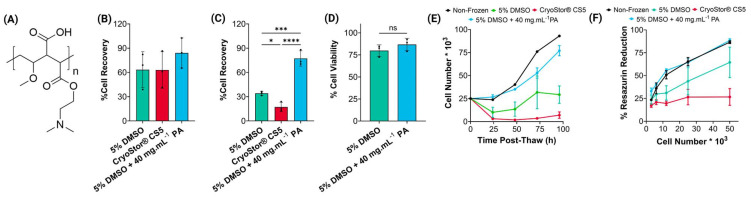
Post-thaw evaluation of THP-1 cryopreservation in cryovials. (A) Structure of cryoprotective polyampholyte;^31^ (B) 0 h post-thaw percentage cell recovery; (C) 24 h post-thaw percentage cell recovery; (D) 24 h post-thaw percentage cell viability. (E) 96 h post-thaw growth curve. (F) Metabolic activity (24 h post-thaw) using the resazurin reduction assay across different cell densities. (B, C, D and E) Were measured using the trypan blue exclusion method. Data expressed as mean ± SD. For all conditions, 3 biological repeats and 2 technical replicates each were evaluated, except for non-frozen control (E), where the results of technical replicates are reported. Statistical significance was determined using either a one-way ANOVA with Tukey *post-hoc* test (B and C) or unpaired two-tailed *T*-test (D).

Firstly, we explored THP-1 cryopreservation in cryovials (1 mL) which is the most widely used cryopreservation format. Three different cryopreservation solutions were evaluated: 5% DMSO + 20% FBS, a common formulation in academic laboratories, abbreviated to 5% DMSO; the commercially available CryoStor® 5 (CS5), also containing 5% DMSO; and 5% DMSO + 20% FBS supplemented with 40 mg mL^−1^ polyampholyte (poly(vinyl ether-*alt*-maleic acid mono(dimethylamino ethyl)ester)), which will be henceforth abbreviated to 5% DMSO + PA (Fig. 1A). Cells were cryopreserved at a cell density of 1 × 10^6^ cells mL^−1^, cooled at −1 °C per minute and stored at −80 °C for 24 hours before thawing.

Immediately post-thaw (Fig. 1B) total recovery was determined by trypan blue exclusion assay. There were no significant differences between the conditions evaluated, averaging a mean cell recovery above 60%. The 5% DMSO + PA condition showed a higher cell recovery (84%), but this difference was not statistically significant. After 24 h post-thaw, cell recovery was drastically reduced for the 5% DMSO and CS5 conditions (34 and 17%, respectively) compared to the immediate post-thaw values, whilst 77% of cells cryopreserved using 5% DMSO + PA presented intact cell membranes (Fig. 1C). Whilst 0 h post-thaw is a useful timepoint for screening, there is insufficient time for apoptosis (delayed-onset-cell-death) to set in, leading to over-estimated cell recovery.^38–40^ Cell viability (trypan blue exclusion) was not significantly different between the 5% DMSO and 5% DMSO + PA conditions (Fig. 1D). This highlights the importance of evaluating cell recovery 24 h post-thaw, as it is not influenced by the ratio of live to dead cells (*i.e.* viability), but rather, the number of cells recovered post-thaw, preventing false positives.

To use THP-1 cells in subsequent assays, it is essential they maintain a standard growth pattern. Cell numbers were monitored over 96 h post-thaw to check their growth and doubling times (Fig. 1E). Cryopreservation with 5% DMSO + PA presented growth curves closest to fresh cells, with doubling times of 47.4 and 55.9 ± 13 h in the non-frozen control and 5% DMSO + PA conditions respectively. In contrast, in the 5% DMSO and CS5 conditions, cell number decreased during the first 24 h post-thaw, with slow, variable cell growth and no overall doubling of the population within the 96 h assessed. These observations are presumably due to apoptosis onset (especially during the first 24 hours) and shows that although cells can look healthy immediately post-thaw, extended culture is required for validation.

Testing cell health using a single assay could deliver results about the effectiveness of a cryoprotectant, as no single parameter is truly representative of viability.^41^ Additionally, it is essential to ensure the results from biochemical assays are comparable to those obtained using non-frozen cells. Therefore, after performing a serial dilution of non-frozen and cryopreserved cells at the 0 h post-thaw timepoint, the percentage resazurin reduction was quantified after a 24 h culture period. This measured whether assay outcomes are comparable at several cell densities (Fig. 1F). The resazurin reduction trend was nearly identical between 5% DMSO + PA and non-frozen cell controls, highlighting how cryopreserved cells are suitable for screening. Cells cryopreserved using 5% DMSO showed reduced metabolic activity, but CS5 did not show increase of metabolic activity at higher cell densities, further suggesting that cells are undergoing apoptosis in conditions without the polyampholyte.

### Addition of a chemical ice nucleator improves recovery and reduces variability after THP-1 cryopreservation in 96-well plates

Following successful cryopreservation of THP-1 cells in cryovials using 5% DMSO + PA, the 96-well plate format was tested. Banking cells in multi-well plates can accelerate assays by removing culturing, splitting and quality control steps. In 24 or 12 well plates, where the liquid volume and surface area are relatively large, polyampholytes have been shown to increase cell recovery to approximately 100% for adherent cell lines.^31,42^ The transfer to 96-well plates is far more challenging.^43^ Due to the low volumes, cryoprotectant solutions supercool far below the equilibrium freezing point, which reduces cell recovery by increasing the amount of (fatal) intracellular ice formation.^23,44,45^ Furthermore, supercooling is stochastic, so each well freezes at a different temperature, leading to well-to-well variability.^23^ To address this, the 5% DMSO + PA formulation was additionally supplemented with pollen-derived macromolecules as a chemical ice nucleator (see methods). This emerging nucleator is not fully characterised, but is the only soluble polymeric ice nucleator used in cryobiology.^25,26,28,32,46^ This formulation will be referred to as 5% DMSO + PA + IN.

**Fig. 2:**
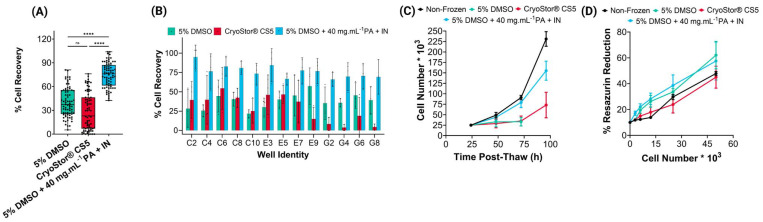
Post-thaw evaluation of THP-1 cryopreservation in 96-well plates. (A) 24 h post-thaw percentage cell recovery; (B) well-dependent percentage cell recovery results; (C) 96 h post-thaw growth curve; (D) metabolic activity measured using the resazurin reduction assay 24 h post-thaw across different cell densities. (A, B and C) Were measured using the trypan blue exclusion assay. (A) Boxplot shows the median, lower and upper quartiles and whiskers represent the range. (B, C and D) Data represented as mean ± SD. 3 biological and 2 technical replicates were performed except for (A) where inter- and intra-plate variability is reported. Statistical significance was determined using a Kruskal–Wallis test with Dunn *post-hoc* (A).

Cell recovery was measured 24 h post-thaw, as above. To quantify spread of the dataset, the interquartile range (IQR), together with the lower (Q1) and upper quartiles (Q3) are reported. The results (Fig. 2A) showed a significantly higher cell recovery when using 5% DMSO + PA + IN, with a median of 76% of cells showing intact membranes post-thaw (IQR: 25%, Q1–Q3: 62–87%). For cells cryopreserved with 5% DMSO the median recovery was 37% (IQR: 29%, Q1–Q3: 26–55%) and using CS5, 30% (IQR: 44%, Q1–Q3: 8–52%). 5% DMSO + PA + IN provides a significantly higher cell recovery and smaller spread of the data, as determined by the IQR values. Additionally, a multi-well plate assay must have a consistent and accurate number of cells between different wells (*i.e.* technical replicates) and plates (*i.e.* biological replicates), therefore, cell recovery was evaluated at selected wells and compared between the different cryoprotectants. For the 5% DMSO + PA + IN formulation, a median cell recovery of 77% was observed with minimal variance within different wells across the plate (65–95%). In contrast, the 5% DMSO condition ranged between 57% average recovery in well E9 to less than 22% in well C10 (Fig. 2B and ESI Fig. 1†). In the CS5 dataset, this effect is even more pronounced, with values ranging from 4% to 54% average recovery. This shows that using standard cryopreservation formulations, results from cryopreserved multi-well plates could not be trusted, as the post-thaw cell number variation would outweigh any assay results.

When evaluating cell growth over 96 h post-thaw, but after seeding cells 24 h post-thaw to reduce the impact of delayed-onset-cell-death in the results (Fig. 2C), a faster doubling time was observed than when using cryovials. The 5% DMSO + PA + IN condition presented a growth rate of 30.3 ± 7.1, comparable to the non-frozen control (25.0 ± 1.5 h). In contrast, the CS5 condition presented a remarkably slow growth rate (55.1 ± 21.13 h) and the DMSO condition did not double within the 96 h assessed. These conditions showed a high standard deviation, evidencing the challenge of uncontrolled ice-nucleation. The percentage metabolic activity after 96-well plate cryopreservation (Fig. 2D) did not significantly change between non-frozen and frozen conditions with different cryoprotectants, a superior result compared to vial cryopreservation. This might be due to reduced cell stress during the cryopreservation/thawing procedures (less volume and hence faster thaw), or less cells lost due to transfer. Cell numbers were seeded post-thaw, individually for each cryoprotectant condition, removing the influence from low cell recovery.

### Macromolecular cryoprotectants induce cell dehydration and reduce intracellular ice formation and apoptosis

The above data showed that in some conditions there were lower cell numbers after 24 h than immediately post-thaw. This suggests the onset of apoptosis (programmed cell death), which can take 24–48 h to cause cell death, as previously reported for primary monocytes.^20,47^ Apoptosis was therefore measured using annexin V–FITC and Propidium Iodide (PI) using flow cytometry. One of the first markers exhibited by cells undergoing early apoptosis is the externalisation of phosphatidylserine, usually found in the inner plasma membrane, which annexin V binds to. As apoptosis progresses, membrane integrity is compromised, allowing entry of PI into the cells and confirming cellular death, staining for both annexin V and PI.^48^

**Fig. 3:**
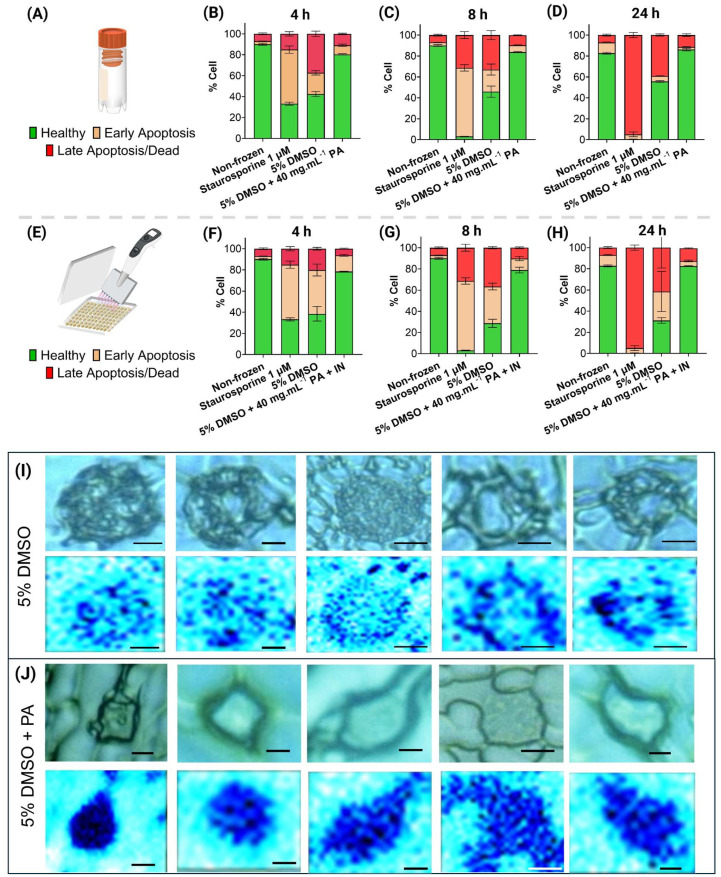
Macromolecular cryoprotectant effect on apoptosis and intracellular ice formation. Panels (A–D) show apoptosis analysis after vial-based cryopreservation; (F–H) shows apoptosis analysis after 96-well plate cryopreservation. Three different cryoprotectants were assessed: 5% DMSO, 5% DMSO + PA (cryovials, A) or 5% DMSO + PA + IN (96-well plates, E), and two controls: non-frozen negative and staurosporine-treated (1 μM, 4 h) positive controls (ESI Fig. 6†). Apoptosis assessed using annexin V/PI assay using flow cytometry. Results represented as mean ± SD, and 3 biological and 3 technical replicates were performed. (I and J) Raman cryomicroscopy of cells cryopreserved with 5% DMSO (I) or 5% DMSO + PA (J). Brightfield images and heatmaps rendered from characteristic spectral signals of ice (light blue) and water (dark blue). Scale bar: 5 μm.

Focusing on vial cryopreservation outcomes at 4 and 8 h post-thaw (Fig. 3A–D and ESI Fig. 2A†), 43% of cells cryopreserved with 5% DMSO were viable, 20% of cells showed signs of early apoptosis (annexin V–FITC positive), and around 30% of the remaining were late apoptotic/dead, (positive for both annexin V and PI). As apoptosis is time dependent,^49^ this observation at the 4 h post-thaw timepoint suggests that cell death may not be due to apoptosis, but rather due to biophysical damage to cells during cryopreservation. This highlights the importance of performing timepoint analysis of apoptosis to confirm the mechanism of cell death. For cells cryopreserved with 5% DMSO + PA, the percentage of cells undergoing both early and late apoptosis was below 10%, and 80% of cells were viable. These results at an early time-point suggest that apoptosis was decreased relative to DMSO-only, and the low percentage of late apoptotic/dead cells support superior preservation of cellular integrity compared to 5% DMSO-only. After 24 h, the 5% DMSO condition showed an increase of around 10% late apoptotic/death cells, strongly suggesting that cells previously detected as undergoing early apoptosis have completed apoptosis. Some cells remain in the early apoptosis phase, suggesting THP-1 cells might take longer than 24 h for apoptosis completion, as reports suggest that some cell types undergo apoptosis even after 48 h post-thaw.^50^ The results from the 5% DMSO-only condition agree with those from cryopreserved monocytes in previous studies, with around 20–30% showing signs of apoptosis.^51^

When considering 96-well plate cryopreservation outcomes (Fig. 3E–H and ESI Fig. 2B†), at the 4 h timepoint, around 40% of cells cryopreserved with 5% DMSO-only are showing early apoptosis and around 20% are late apoptotic/dead. The percentage of early apoptotic cells is higher than observed after cryovial cryopreservation, whilst the percentage of late apoptotic/dead cells is lower. This suggests less biophysical damage during cryopreservation but an increased incidence of apoptosis. By the 8 h timepoint, there was a 20% increase in late apoptotic cells, evidencing that some cells lost membrane integrity, whilst the number of annexin V positive cells continued to decrease after 24 h, and both viable and late apoptotic cells slightly increased. In contrast, cells cryopreserved with 5% DMSO + PA + IN showed around 80% viability through the total post-thaw period, with the percentage early and late apoptotic cells remaining relatively stable throughout, suggesting that apoptosis was not significantly impacting cell viability.

Previous work suggests that polyampholytes act as cryoprotectants by facilitating cellular dehydration and reducing intracellular ice formation.^33,34^ To assess the mechanism of action of these macromolecular cryoprotectants, THP-1 cells were treated with either 10% DMSO (ESI Fig. 3†) 5% DMSO or 5% DMSO + PA and cooled at −1 °C per minute using a cryo-stage. Then, Raman cryomicroscopy was performed (Fig. 3I and J) to visualise intracellular ice formation based on the distinct Raman spectra of ice and water.^52,53^ Five individual cells, and their respective ice/water heatmaps are shown per condition, enabling the estimation of relative ice formation per cell. In the 5% DMSO condition, 47.5–68.4% ice was found (ESI Fig. 4†), appearing as light-blue pixels (Fig. 3J). In contrast, only 0.1–2.8% of the cells displayed intracellular ice (*i.e* vitrifed) during cryopreservation with 5% DMSO + PA (ESI Fig. 5†) appearing as dark blue in Fig. 3J. This dramatic decrease in intracellular ice formation after addition of the polyampholyte to the cryopreservation media confirms its role in cellular dehydration and intracellular ice prevention.

### Macromolecular cryoprotectants improved post-thaw differentiation of THP-1 into a macrophage-like phenotype

**Fig. 4:**
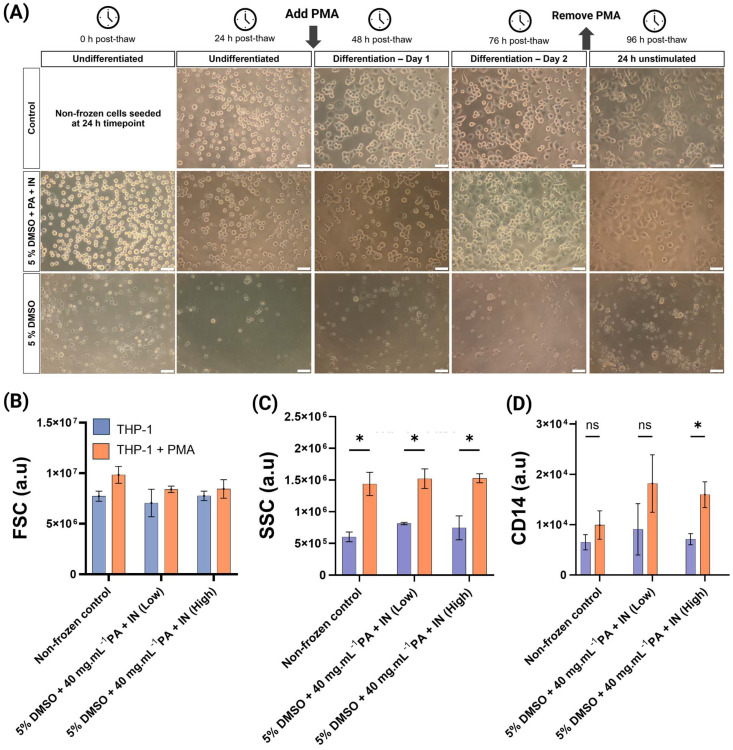
Characterisation of post-thaw THP-1 differentiation into macrophages. (A) Phase contrast microscopy of cryopreserved THP-1 cells in 96-well plates over 96 h post-thaw after differentiation using PMA. Representative images from 3 biological replicates, each with 3 technical replicates. Non-frozen control included for comparison. Scale bar: 50 μm; (B) median forward scatter (FSC); (C) median side scatter (SSC); (D) median fluorescence intensity of CD14–PerCP Cy5.5, see ESI Fig. 8.† For (B, C and D), results were measured using flow cytometry and reported as median ± SD and 3 biological replicates, each with 3 technical replicates are reported. Statistical significance was determined using multiple unpaired *T*-test (with Holm-Sidak correction) between the THP-1 and THP-1 + PMA conditions.

THP-1 cells are primarily used to be differentiated into macrophages, making them essential for both monocyte and macrophage biology and immunology.^9^ A widely used differentiation method is applying phorbol 12-myristate 13-acetate (PMA). PMA stimulates the protein kinase C, MAPK and NF-κB pathways, crucial in monocyte to macrophage differentiation.^54–56^ THP-1 cryopreservation in 96-well plate format could remove the need to grow, culture and plate the cells post-thaw, by just thawing, waiting 24 h and inducing differentiation. THP-1 cells were cryopreserved in 96-well plates at a cell density of 50 000 cells per well using the 5% DMSO and 5% DMSO + PA + IN formulations. After a 24 h post-thaw culture, cells were treated with 130 nM PMA for 48 h and were either cultured at the same cell density as cryopreserved, or wells were counted and pooled to represent the “ideal” cell density of 100 000 per well. This was performed to remove the effects that low cell density after poor cell recovery could have in post-thaw survival and differentiation. For the 5% DMSO-only condition there was near-zero cell recovery or differentiation regardless of cell density. The undifferentiated condition presented with more “granular” and irregularly shaped cells, compared with the non-frozen control or the 5% DMSO + PA + IN condition. After treatment with PMA, microscopy revealed reduced cell size, abnormal and irregular shape (Fig. 4A) and flow cytometry recognised most events as debris (ESI Fig. 7†). Therefore, the 5% DMSO condition was not included in further analysis.

Differentiated THP-1 cells are characterised by an increase in forward scatter, or FSC –proportional to cellular diameter– and side scatter (SSC) which measures internal cellular complexity.^5^Fig. 4(B) showed increased FSC after differentiation, especially in the non-frozen control. This difference was not significantly different in any condition. Similar studies differentiating THP-1 cells using PMA report no differences in FSC, or slight differences only.^57,58^ SSC measurements (Fig. 4C) showed a clear and significant increase after THP-1 treatment with PMA. There were no significant differences between control and cryopreserved conditions, confirming successful differentiation. An additional marker of THP-1 differentiation into a macrophage-like phenotype is increased CD14 cell surface expression.^54,59^ There was a slight increase in CD14 fluorescence in the non-frozen control after differentiation but was not significant. In the previously cryopreserved cells, the median fluorescence intensity after PMA treatment increased relative to the non-frozen control, and in high cell culture density, the differences were significant. The sample size did not allow for the detection of a significant increase in CD14, but the consistent fluorescence increase was encouraging. It is important to highlight that the culture conditions,^60^ differentiation inducer and concentration affect the differentiation process and complicates comparisons. Differentiation using calcitriol reportedly increases CD14 expression more than PMA.^54^ Additionally, there are conflicting reports, as THP-1 immunofluorescence and flow cytometry have previously shown a reduction in CD14 fluorescence after differentiation, rather than an increase.^61,62^

## Conclusions

Here we demonstrate the addition of macromolecular cryoprotectants improved THP-1 post-thaw outcomes compared to the gold-standard of DMSO. The use of cryoprotective polyampholyte is shown to reduce intracellular ice formation (*i.e.* promoting a desirable glassy state) using Raman cryomicroscopy, providing a mechanism of action. Chemically induced ice nucleation enabled THP-1 cryopreservation in 96-well plates to allow direct use from the freezer with no additional handling steps. This cryoprotectant combination enabled successful differentiation of THP-1 cells into macrophages, a significant improvement relative to DMSO-only formulations. The combination of the two extracellular macromolecular cryoprotectants to reduce intracellular ice, as well as well-to-well variation associated with supercooling may bring benefits to other monocyte cells where traditional solutions give low recovery rates, but will require more study. The improved cell health and minimal apoptosis will remove key bottlenecks in monocyte and macrophage biology by accelerating data collection and enabling ‘assay-ready’ cells for high-throughput screening.

## Author contributions

Conceptualization, methodology, N. G.-M., R. T., A. B., A. N., M. I. G.; investigation and analysis, N. G.-M., R. T., A. B., A. N., A. I.; writing original draft, N. G.-M., M. I. G.; writing – review & editing, all authors; funding acquisition, M. I. G.

## Conflicts of interest

M. I. G. is a director and shareholder of Cryologyx Ltd. R. T. is currently employed by Nuclera Ltd. All other authors have no conflicts to declare.

## Supplementary Material

LP-003-D5LP00131E-s001

## Data Availability

The authors declare that the data supporting the findings of this study are available within either the paper or the ESI.† The code used to calculate percentage of intracellular ice is included in ESI Methods 1.†
