# Supplementary material for: Cryopreservation and post-thaw differentiation of monocytes enabled by macromolecular cryoprotectants which restrict intracellular ice formation
Source: RSC Appl Polym. 2025 Jun 9;3(4):990–1001. doi: 10.1039/d5lp00131e (PMC12147442; doi:10.1039/d5lp00131e)
Supplement: LP-003-D5LP00131E-s001 [file LP-003-D5LP00131E-s001.pdf]

Supplementary Information (SI) for RSC Applied Polymers.
This journal is © The Royal Society of Chemistry 2025

Supplementary information for:

Cryopreservation and Post-Thaw Differentiation of Monocytes Enabled by Macromolecular Cryoprotectants which Restrict Intracellular Ice Formation

Natalia Gonzalez-Martinez,[a,b,c] Ruben M. F. Tomás,[d] Akalabya Bissoyi,[b,c] Agnieszka Nagorska,[d] Alexandru Ilie,[b,c] and Matthew I. Gibson[b,c,*]

[a]Department of Chemistry, University of Warwick, Gibbet Hill Road, CV4 7AL, Coventry, UK.

[b]Manchester Institute of Biotechnology, University of Manchester, 131 Princess Street, Manchester M1 7DN, U.K.

[c]Department of Chemistry, University of Manchester, Oxford Road, Manchester M13 9PL, U.K.

[d]Cryologyx Ltd, Venture Centre, University of Warwick Science Park, Coventry CV4 7EZ, U.K.

[*]Corresponding Author Email, matt.gibson@manchester.ac.uk

Supplementary information

Supplementary Method 1: Threshold-based segmentation and Monte Carlo simulations to quantify intracellular ice

```
from PIL import Image

import numpy as np

from skimage import measure

import matplotlib.pyplot as plt


def calculate_red_and_combined_blue(image_array, size_threshold=50, iterations=50, threshold_variation=10):
    """
    Calculate the area percentages for red and combined blue (light + dark blue) regions in an image,
    with statistical analysis using Monte Carlo simulation.
    """
    base_red_thresh = {"r_min": 200, "g_max": 100, "b_max": 100}
    base_blue_thresh = {"r_max": 100, "g_max": 150, "b_min": 100}


    red_percentages = []
    blue_percentages = []


    for _ in range(iterations):
        red_thresh = {
```

```
        "r_min": base_red_thresh["r_min"] + np.random.randint(-threshold_variation, threshold_variation),
        "g_max": base_red_thresh["g_max"] + np.random.randint(-threshold_variation, threshold_variation),
        "b_max": base_red_thresh["b_max"] + np.random.randint(-threshold_variation, threshold_variation),
    }
    blue_thresh = {
        "r_max": base_blue_thresh["r_max"] + np.random.randint(-threshold_variation, threshold_variation),
        "g_max": base_blue_thresh["g_max"] + np.random.randint(-threshold_variation, threshold_variation),
        "b_min": base_blue_thresh["b_min"] + np.random.randint(-threshold_variation, threshold_variation),
    }

    red_mask = (image_array[:, :, 0] >= red_thresh["r_min"]) & (image_array[:, :, 1] <= red_thresh["g_max"]) & (image_array[:, :, 2] <= red_thresh["b_max"])
    combined_blue_mask = (image_array[:, :, 0] <= blue_thresh["r_max"]) & (image_array[:, :, 1] <= blue_thresh["g_max"]) & (image_array[:, :, 2] >= blue_thresh["b_min"])

    red_area = np.sum(red_mask)
    combined_blue_area = np.sum(combined_blue_mask)
    total_area = red_area + combined_blue_area
```

```
    if total_area > 0:
        red_percentages.append((red_area / total_area) * 100)
        blue_percentages.append((combined_blue_area / total_area) * 100)

  results = {
    "red_mean": np.mean(red_percentages),
    "red_std": np.std(red_percentages),
    "blue_mean": np.mean(blue_percentages),
    "blue_std": np.std(blue_percentages),
  }

  return results

def visualize_with_text_on_top(image_array, results, output_file=None):
  """
  Visualize the image with borders and statistics displayed as text on top.
  """
  red_thresh = {"r_min": 200, "g_max": 100, "b_max": 100}
  blue_thresh = {"r_max": 100, "g_max": 150, "b_min": 100}

  red_mask = (image_array[:, :, 0] >= red_thresh["r_min"]) & (image_array[:, :, 1] <= red_thresh["g_max"]) & (image_array[:, :, 2] <= red_thresh["b_max"])
```

```
    combined_blue_mask = (image_array[:, :, 0] <= blue_thresh["r_max"]) & (image_array[:, :, 1] <= blue_thresh["g_max"]) & (image_array[:, :, 2] >= blue_thresh["b_min"])

    red_contours = measure.find_contours(red_mask.astype(int), 0.5)
    blue_contours = measure.find_contours(combined_blue_mask.astype(int), 0.5)

    fig, ax = plt.subplots(figsize=(8, 8))
    ax.imshow(image_array)
    ax.axis('off')

    for contour in red_contours:
        ax.plot(contour[:, 1], contour[:, 0], color='black', linewidth=1)

    for contour in blue_contours:
        ax.plot(contour[:, 1], contour[:, 0], color='black', linewidth=1)

    # Add text on top of the image
    plt.text(0.5, 1.05,
             f"Red: {results['red_mean']:.2f}% ± {results['red_std']:.2f}% | Blue: {results['blue_mean']:.2f}% ± {results['blue_std']:.2f}%",
             fontsize=12, ha='center', transform=ax.transAxes)

    if output_file:
```

```python
        plt.savefig(output_file, dpi=300, bbox_inches='tight')
    plt.show()


# General workflow
# Replace these variables with your file paths
image_path = "your_image_path_here.jpg"  # Replace with your image path
output_path = "your_output_path_here.jpg"  # Replace with your output file path


# Load the image
image = Image.open(image_path).convert("RGB")
image_array = np.array(image)


# Perform the analysis
results = calculate_red_and_combined_blue(image_array, size_threshold=50, iterations=50, threshold_variation=10)


# Visualize with results on top
visualize_with_text_on_top(image_array, results, output_file=output_path)
```

Supplementary Figures

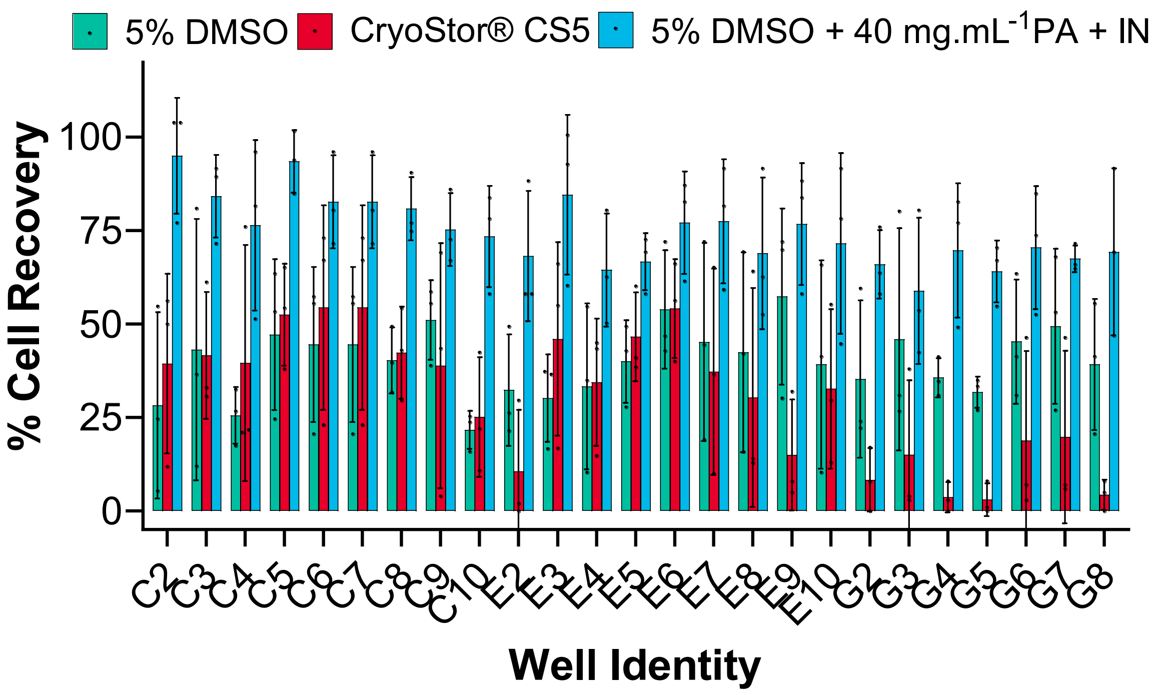

Supplementary Figure 1 Full dataset of well-to-well variability in THP-1 cells cryopreserved in 96-well plates with 3 different cryopreservation formulations. Data was obtained using the Trypan Blue exclusion assay. Data represented as mean ± SD. 3 biological replicates (i.e. 3 different plates) were assessed.

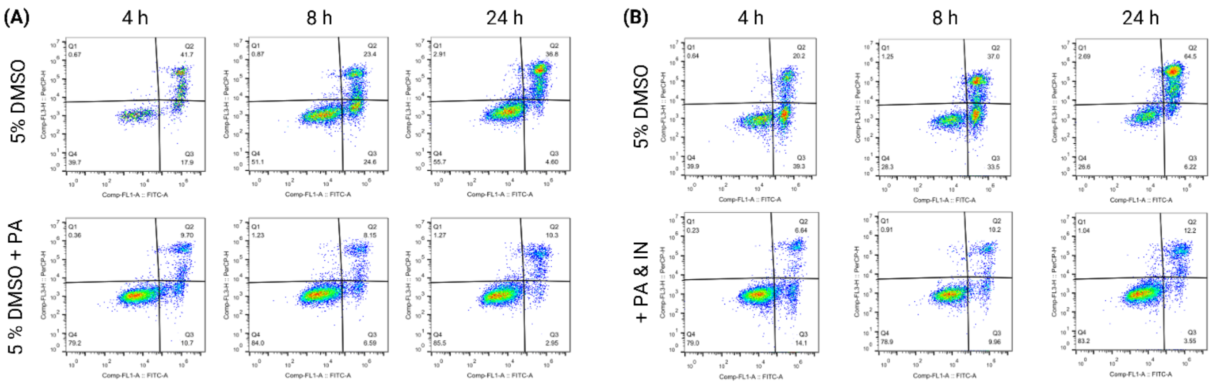

Supplementary Figure 2 Representative dot plots from time-course apoptosis analysis of THP-1 cells after cryopreservation in (A) cryovials or (B) 96-well plates. For (A) cells were cryopreserved with either 5 % DMSO-only or 5 % DMSO + PA. For (B) 5 % DMSO-only and

5 % DMSO + PA + IN were used instead. Analysed using flow cytometry. FITC (x-axis) represents Annexin V-FITC fluorescence and PerCP (y-axis) denotes Propidium Iodide.

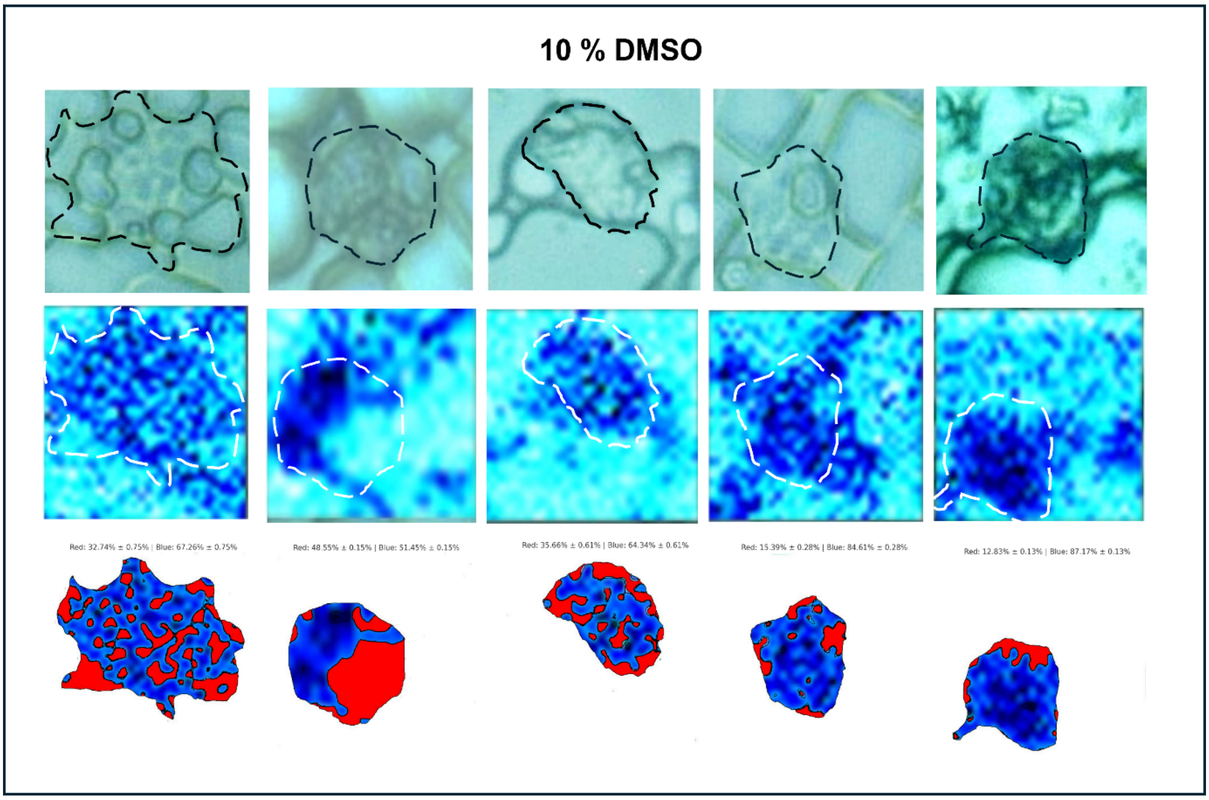

Supplementary Figure 3 Cryo-Raman microscopy of THP-1 cells cryopreserved in 10 % DMSO. The top row shows brightfield microscopy images. The middle row shows heat maps rendered from characteristic Raman spectra signals of ice and water; water is depicted in dark blue and ice in light blue. White dashed line represents the cell boundaries. The bottom row shows threshold-based segmentation and Monte Carlo simulation results, based on the outlined areas, to quantify intracellular ice or water within cells. Ice is depicted in red and water in blue. Analysis performed using Python libraries Pillow, NumPy, scikit-image and MatPlotLib.

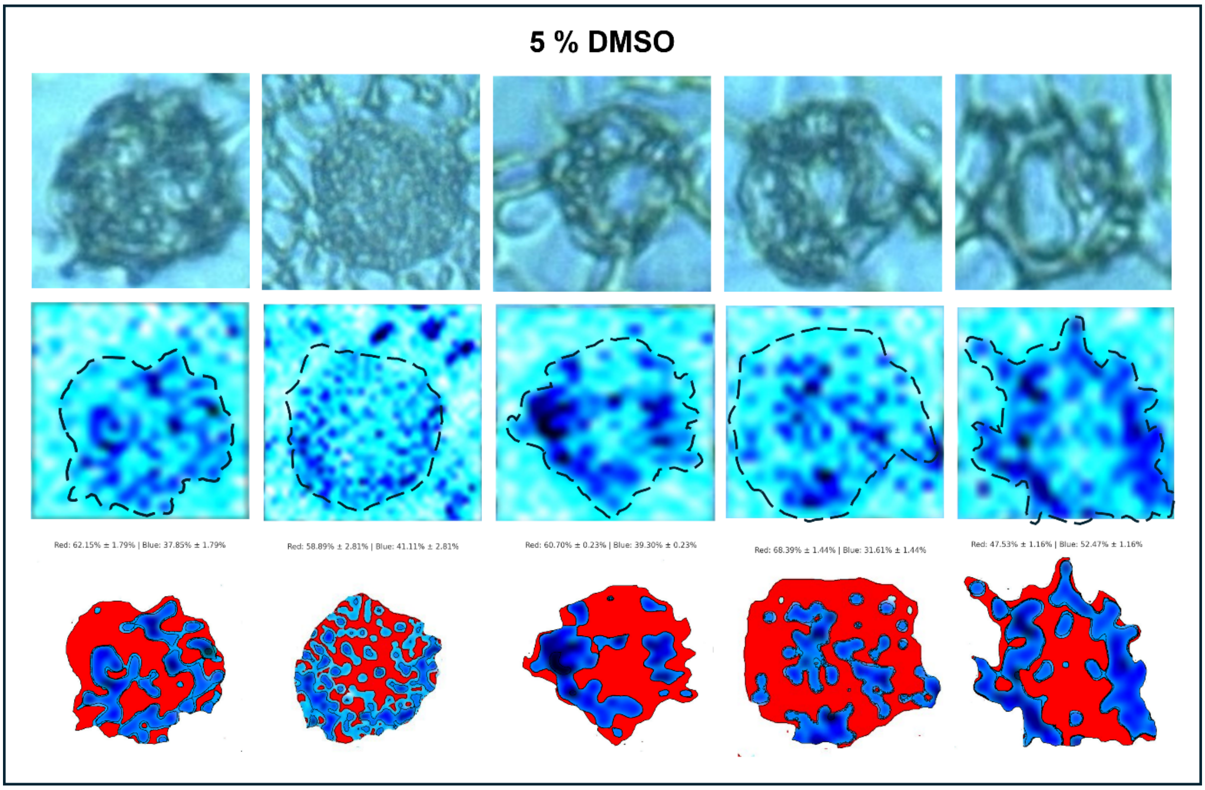

Supplementary Figure 4 Cryo-Raman microscopy of THP-1 cells cryopreserved in 5 % DMSO. The top row shows brightfield microscopy images. The middle row shows heat maps rendered from characteristic Raman spectra signals of ice and water; water is depicted in dark blue and ice in light blue. White dashed line represents the cell boundaries. The bottom row shows threshold-based segmentation and Monte Carlo simulation results, based on the outlined areas, to quantify intracellular ice or water within cells. Ice is depicted in red and water in blue. Analysis performed using Python libraries Pillow, NumPy, scikit-image and MatPlotLib.

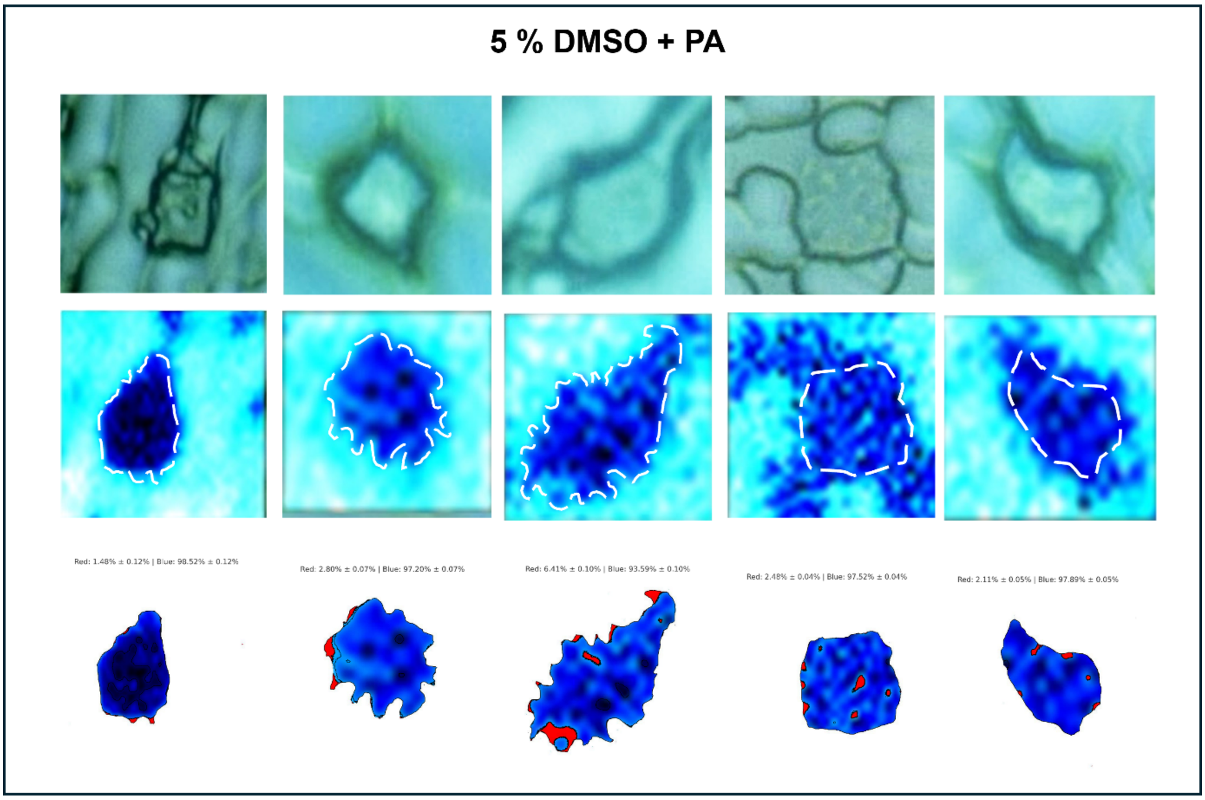

Supplementary Figure 5 Cryo-Raman Spectroscopy of THP-1 cells cryopreserved in 5 % DMSO + 40 mg mL polyampholyte. The top row shows brightfield microscopy images. The middle row shows heat maps rendered from Raman spectra based on characteristic signals of ice and water; water is depicted in dark blue and ice in light blue. White dashed line represents the cell boundaries. The bottom row shows threshold-based segmentation and Monte Carlo simulation results, based on the outlined areas, to quantify intracellular ice or water within cells. Ice is depicted in red and water in blue. Analysis performed using Python libraries Pillow, NumPy, scikit-image and MatPlotLib.

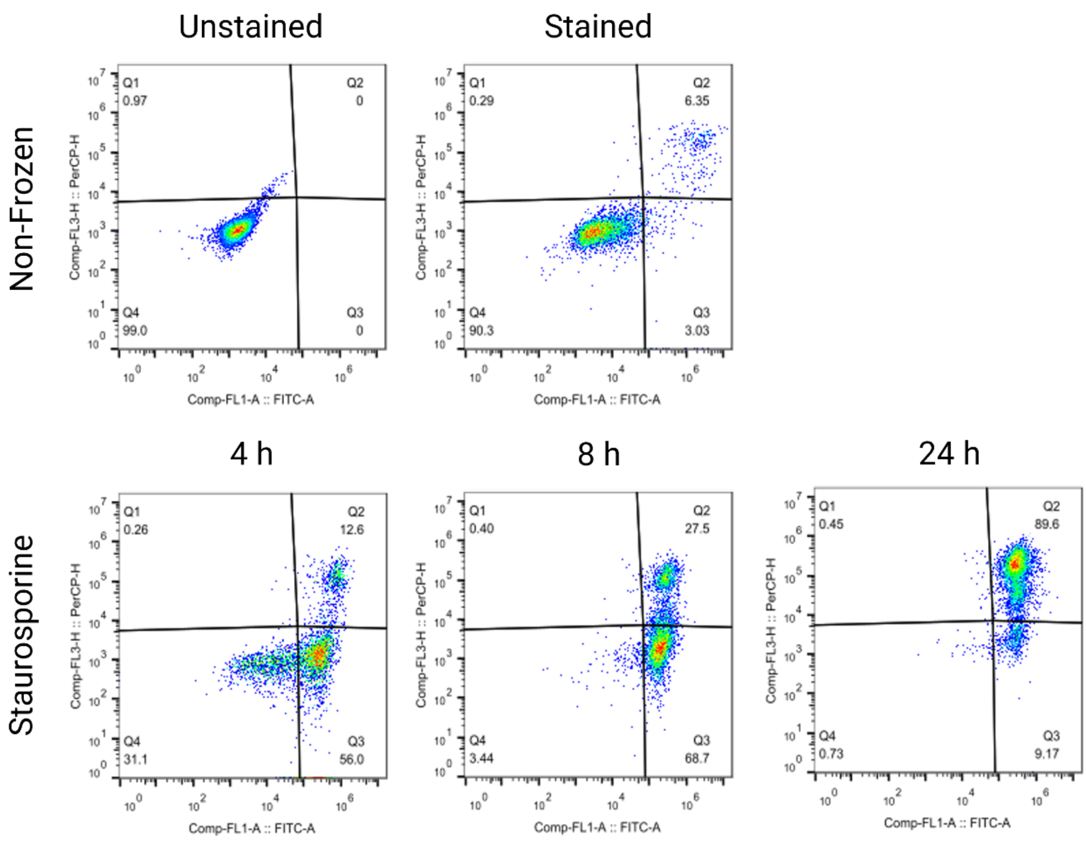

Supplementary Figure 6 Representative dot plots from time-course apoptosis analysis using appropriate assay controls. Non-frozen THP-1 cells served as the negative control of the assay (top row) showing negligible apoptosis. THP-1 cells treated with 1 µM staurosporine for 4 h served as the positive control of apoptosis, showing apoptotic progression after 24 h. Analysed using flow cytometry. FITC (x-axis) represents Annexin V-FITC fluorescence and PerCP (y-axis) denotes Propidium Iodide.

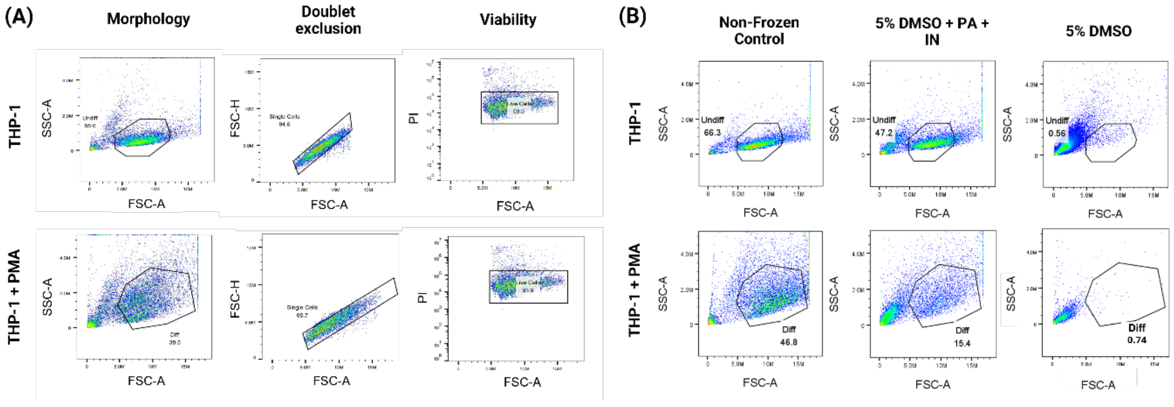

Supplementary Figure 7 Gating strategy and size (FSC) and granularity (SSC) plots following THP-1 differentiation. (A) Initial gating was based in FSC and SSC characteristics, to exclude debris. Doublets were excluded by plotting FSC-A vs FSC-H from this initial gating population. Calculating viability (using PI) verified the initial FSC/SSC gating strategy by backgating to identify and avoiding inclusion of membrane damaged cells. (B) FSC vs SSC flow cytometry plots of non-frozen controls and THP-1 cells cryopreserved with either 5 % DMSO or 5 % DMSO + PA + IN.

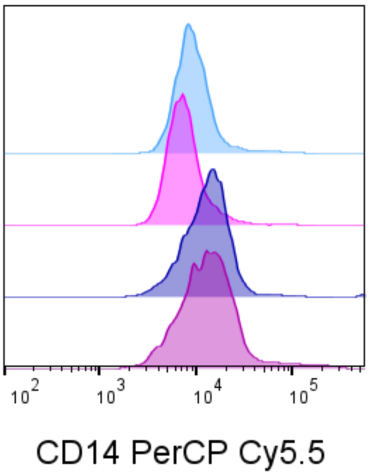

	Sample Name	Subset Name	Count	Median : FL3-A
	A05 CD14_undiff_control_1.fcs	Single Cells	6905	8721
	C08 CD14_undiff_cryoS_high_2.fcs	Single Cells	6376	7103
	A07 CD14_diff_control_1.fcs	Single Cells	6690	12982
	B10 CD14_diff_cryoshiel_L_1.fcs	Single Cells	2445	12928

Supplementary Figure 8 Histogram depicting representative raw median CD14 fluorescence intensities in undifferentiated and differentiated THP-1 cells. Non-frozen control shown as light (undifferentiated) and dark (PMA-treated) blue. Cells cryopreserved with 5 % DMSO + PA shown as pink (undifferentiated) and purple (PMA-treated).
